# Controlled Defect Based Ultra Broadband Full-sized Metamaterial Absorber

**DOI:** 10.1038/s41598-018-27920-1

**Published:** 2018-06-22

**Authors:** Manh Cuong Tran, Dinh Hai Le, Van Hai Pham, Hoang Tung Do, Dac Tuyen Le, Hong Luu Dang, Dinh Lam Vu

**Affiliations:** 1 Faculty of Physics, Hanoi National University of Education, 136 Xuan Thuy, Cau Giay, Hanoi Vietnam; 2 Institute of Materials Science, Vietnam Academy of Science and Technology, 18 Hoang Quoc Viet, Hanoi, Vietnam; 3 Institute of Physics, Vietnam Academy of Science and Technology, Hanoi, Vietnam; 4 Department of Physics, Hanoi University of Mining and Geology, 18 Pho Vien, Bac Tu Liem, Hanoi Vietnam

## Abstract

Metamaterial full-sized absorber structures are numerically and experimentally investigated in GHz region and then examined in THz frequency. By manipulating monitoring the number and the position of the defect elements in conventional unit cells, the optimal integrative absorber structures are generated. The proposed structures provide an ultra-broadband absorbance in the operating frequency. The good agreement between simulation, measurement and theoretical analysis is observed with a 5 GHz-bandwidth corresponding to the absorption of 95%. In particular, we extrapolate the concept to THz region and demonstrate that, the method can be applied to increase the bandwidth of the metamaterial absorber to 5 THz, while maintaining the other characteristics. This structure can be applied to improve the performance of telecommunication systems such as micro-antenna, micro-electromagnetic transmitters and apply to imaging and sensing fields.

## Introduction

Since the first irrationally postulation by Veselago in 1968^1^, and the prototypical experiment investigated by Smith *et al*. in 2000^2^, Metamaterial (MM), an artificial electromagnetic composite material, has emerged muscularly in the last decade. Due to their extraordinary electromagnetic features, MMs have played a principal role in various fields of application such as electrical engineering^3–5^, energy^6,7^, health science^8^, and environment^9^. One of the most notable and attractive properties of this man-made material is perfect absorption.

The first demonstration of metamaterial perfect absorber (MPA) were archetypically proposed by Landy *et al*. in 2008^10^. Since then, various MPAs structures have been extensively investigated^11–16^ with numerous methods, operating in different frequency regions, ranging from MHz to the visible^7,12,14–20^. Such electronic materials are garnering more and more attraction. Especially, broadband MPAs in GHz and THz regions possess many important technological applications in imaging^21^, sensing^22,23^, energy harvesting^24^ or in micro antenna communication^25,26^, which need perfect absorbers to efficiently collect electromagnetic energy. Many efforts to optimize broadband MPAs have been made, such as multi metal-insulator layers^27^, multi resonance mode combination^28^, super unit cell structure^29^. Nevertheless, these methods require complex configuration and optimization. Searching for innovative noncomplex configurations of broadband MPA is therefore always an enormous challenge.

In order to overcome this problem, we propose a novel method that is based on controlled defects. The broadband MPA is created by controlling the number of optimal unit cells and manipulating the defects’ location in the full-sized metamaterial absorber (FSMA). This method is not popular yet is always applied in real technical devices because of their limited geometrical dimensions. In general, the absorption structures are simulated from a unit cell with appropriate boundary conditions supposed to be infinite. With such simulation model, defects cannot be investigated if the structure is periodic. In this work, simulated FSMA resembles the structure in actual size. Open boundary conditions are applied for this configuration. The introduction of controlled defects for the absorption manipulation is therefore feasible and realistic.

The utilized structure is first investigated in the GHz region. The simulation and experiment results show that, when optimized defects are introduced into the structure, the broadband absorption is achieved. Moreover, the proposed structure also achieves polarization insensitive characteristic in the operating frequency due to its symmetric configuration. This technique is then extrapolated to the THz region for THz broadband MPA. The equivalent medium theory (EMT)^30^ is applied to interpret the broadband absorption characteristics of the FSMA and provides a good agreement with the simulation. Interestingly, this method can be employed for various metamaterial full-sized structures to obtain the ultra-broadband absorption in both GHz and THz regions. This technique may initiate a new way of studying broadband metamaterial perfect absorber.

## Absorber Structure in GHz Region

**Figure 1:**
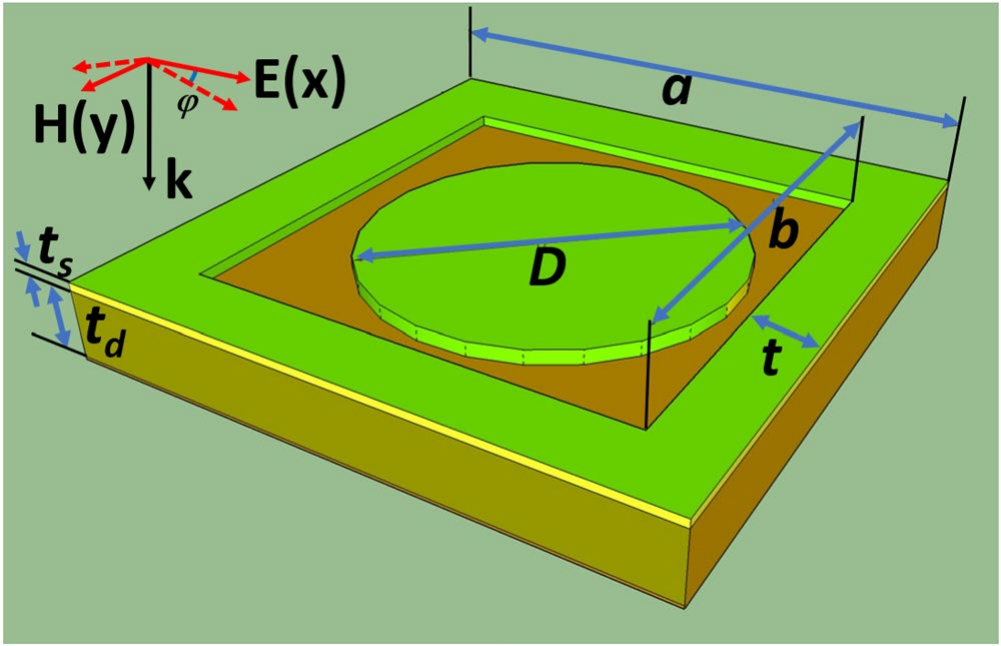
Perspective view of a conventional unit cell with structural parameters.

A full-sized metamaterial structure with integrated defects, which has an ultra-broadband absorbance in GHz frequency region, is investigated. A single unit cell (UC) of the original structure with the geometric parameters is depicted in Fig. 1. We use a 1.5 mm thick (*t*_*d*_) FR4 dielectric substrate which has dielectric constant of 4.3 sandwiched between 0.03 mm thick (*t*_*s*_) copper film on both sides. On the top layer is a 1.25 mm-width (*t*) square ring surround a dish which has the diameter *D* = 3.5 mm. The bottom layer is covered with a full copper film. The copper is modeled as a lossy metal with an electric conductivity σ = 5.82 × 10^7^ S/m. The lattice constant of a single unit cell is *a* = 9 mm; *b* = 6.5 mm. In the simulation, the incident wave is normal to the structure surface, the electric and magnetic fields are parallel to the *x*-axis and the *y*-axis, corresponding to the electric field **E** and magnetic field **H** directions, respectively. The commercial Computer Simulation Technology (CST) Microwave Studio^31^ based on Finite Integration Technique (FIT), is used for the numerical simulation. In the simulation, a waveport is placed upon the structure.

**Figure 2:**
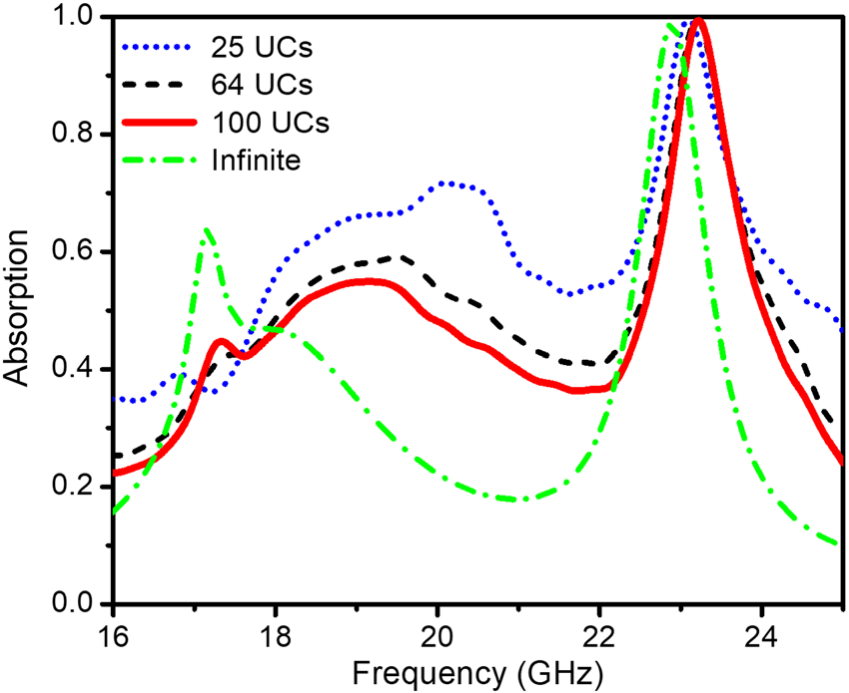
Simulation results: Absorptivity curve of the metamaterial absorber with different number of unit cells.

In order to obtain efficient structure dimensions for further investigation, various full-sized metamaterial structures without defect as 5 × 5, 8 × 8, 10 × 10 UCs are simulated and discussed. The whole considered structure complies with the open boundary conditions in the simulation. Their absorption spectra are compared to that of the case with infinite dimensions (1 unit cell with periodic boundary conditions in the simulation) as illustrated in Fig. 2. Clearly, the main absorption peak is observed at around 23 GHz. The full-sized structure with 10 × 10 unit cells is chosen for the following investigation due to its stable perfect absorption response at the fundamental frequency of around 23 GHz.

**Figure 3:**
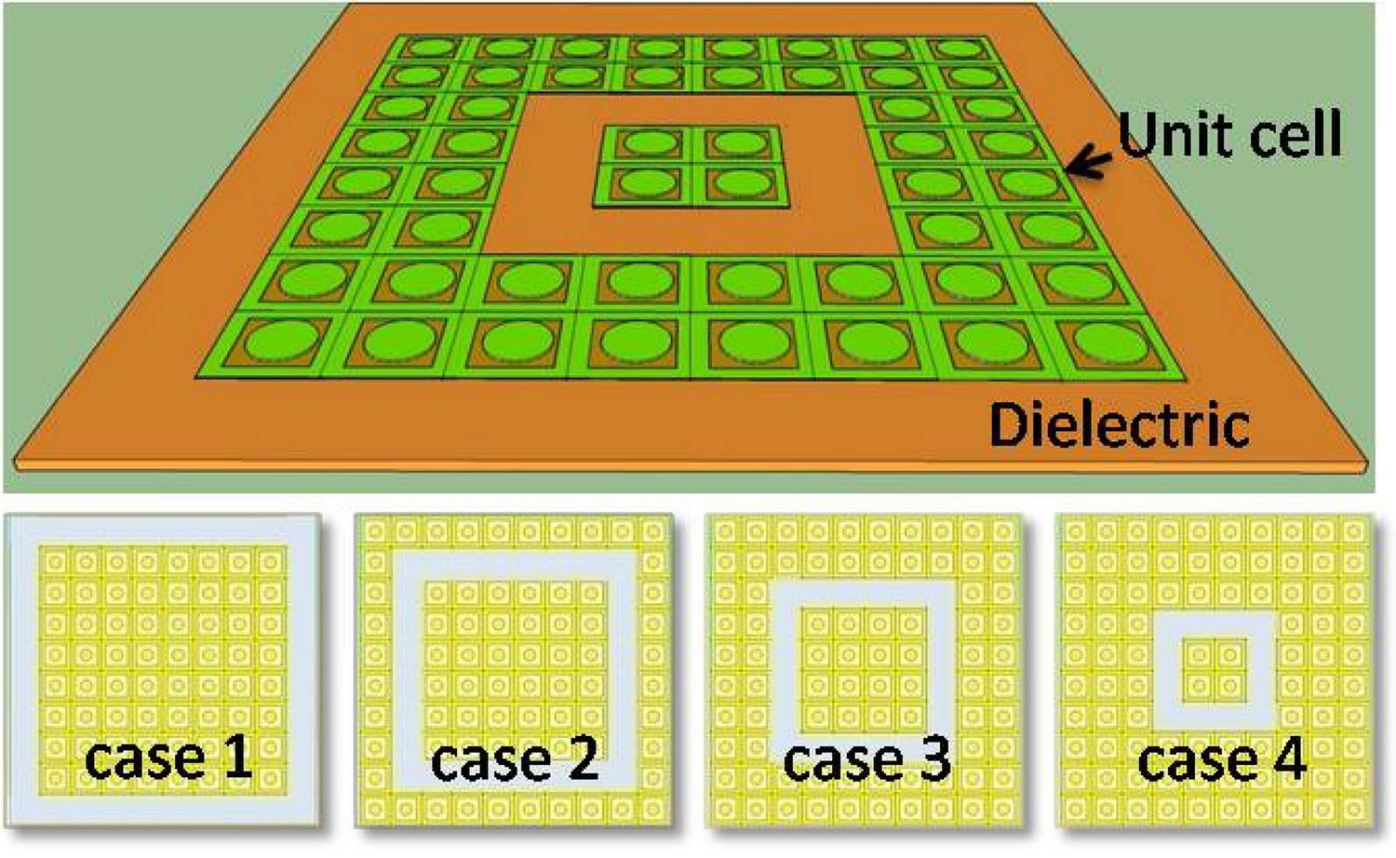
Full-sized combined structure with 100 unit cells (top) and introduced defect cases (bottom).

**Figure 4:**
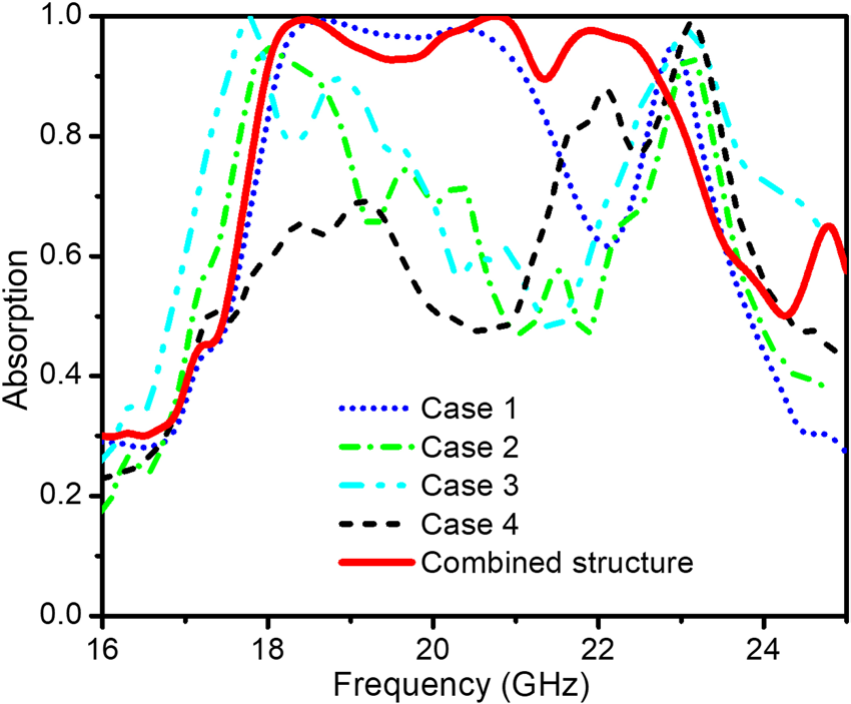
Simulation result: Absorptivity curves of the full-sized metamaterial absorber structure with different defect configurations and the combined structure result.

Next, the so called “defect wall” is introduced by removing one UC rim at a time. The first interested configuration is the outermost rim removed full-sized structure (case 1). Moving stepwise the defect wall inward from case 2 to case 4 generates other configurations as shown in Fig. 3. As mentioned above, with the full-sized structure, the fundamental absorption frequency is centered at around 23 GHz. Interestingly, for case 1, a broadband absorption of about 4 GHz wide is achieved beside the fundamental absorption peak at around 23 GHz (see Fig. 4). Meanwhile, case 4 has a second absorption peak at around 22 GHz next to the fundamental one, this is related to the case of defect wall configuration. Thus, these results suggest an optimal structure combining these two configurations to develop a wider broadband absorption.

Beside 2D picture of these four considered configurations, Fig. 3 also schematically shows the 3D combined metamaterial structure of the cases 1 and 4 with the dimension of 10 × 10 unit cells integrating with the defects and the total surface area is 90 × 90 mm^2^. As predicted, the ultra-broadband of ~5 GHz (from 18–23 GHz) with the absorption of over 95% is achieved in the combined structure (see Fig. 4).

**Figure 5:**
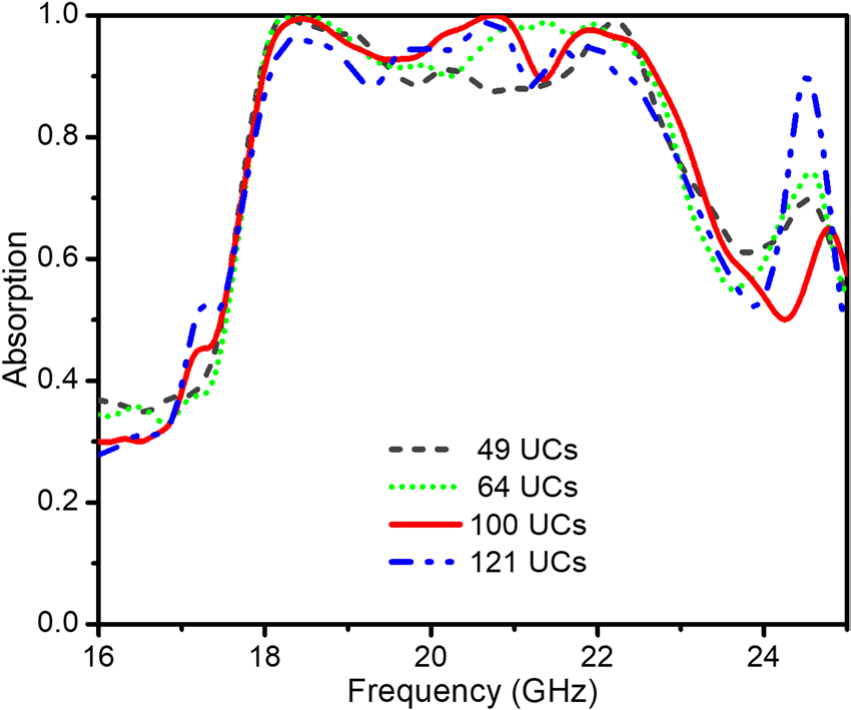
Absorptivity curves of different defect combined full-sized absorber structures with different number of UCs.

In order to verify the validity and flexibility of the proposed method, we apply this technique to other full-sized structures, in which each structure will be introduced two defect walls. Figure 5 indicates the numerically simulated results of various FSMAs consisting of 7 × 7, 8 × 8, 10 × 10 and 11 × 11 UCs. It can be easily pointed out that all these optimal structures own the same broadband absorbance from 18 to 23 GHz with the absorptivity higher than 90%. The presence of the controlled defect wall is critical in increasing the absorption band of the full-sized structure. It is proved that this method can be applied not only to various sizes of FSMA to achieve the broadband absorption spectrum but also to unlock another choice for broadband MPA.

## Broadband Absorption Interpretation Using Equivalent Medium Theory (EMT)

**Figure 6:**
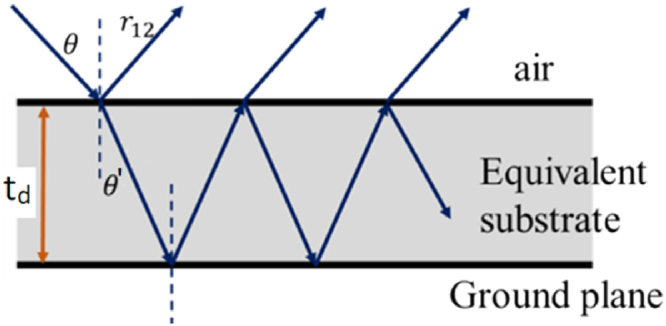
The effectively equivalent medium of the full-sized metamaterial absorber.

To understand more the broadband characteristic of the structure, we employ the equivalent medium theory to obtain the absorption spectra (see Fig. 6). Here, the calculated broad absorbance and the effectively constitutive parameters such as the permittivity (*ε*_*eff*_) and permeability (*μ*_*eff*_) are retrieved from the reflection *S*_11_ and the transmission *S*_21_, which detemine by the numerical simulation^32,33^. The effective impedance and the refractive index of the homogenous and isotropic effective medium are related to *S*_11_ *and S*_21_ by^30^:

1 $${Z}_{eff}=\sqrt{\frac{{\mu }_{eff}}{{\varepsilon }_{eff}}}=\pm \sqrt{\frac{{(1+{S}_{11})}^{2}-{S}_{21}^{2}}{{(1-{S}_{11})}^{2}-{S}_{21}^{2}}}$$

2 $$n=\frac{1}{{k}_{0}d}\{[[\,\mathrm{ln}({e}^{in{k}_{o}d})]^{\prime\prime} +2m\pi ]-i[\mathrm{ln}({e}^{in{k}_{o}d}]^{\prime} \}$$

where ${\varepsilon }_{eff}=\frac{n}{{Z}_{eff}}$, *μ*_*eff*_ = *n*.*Z*_*eff*_ and *m* is an integer corresponding to the branch index *n*′; *k*_0_ is the wave number in the free space. The exponential factor in eq. (2) is related to the S-parameters by

3 $${e}^{in{k}_{0}d}=X\pm \sqrt{1-{X}^{2}}$$

where

4 $$X=\frac{1}{2{S}_{21}(1-{S}_{11}^{2}+{S}_{21}^{2})}$$

with the copper plate at the bottom layer, the transmission coefficient *S*_21_ is close to zero. As a result, the values of the refrective index *n* are very sensitve to small variations of *S*_21_, which could lead to a failure of the retrieval method^33^. However, a small *S*_21_ has little effect on the retrivial of *Z*_*eff*_ as shown by the calculation of the derivative of ${Z}_{eff}^{2}$ with respect to *S*_21_,

5 $$\frac{\partial {Z}_{eff}^{2}}{\partial {S}_{21}}=\frac{8{S}_{21}{S}_{11}}{[{(1-{S}_{11})}^{2}-{S}_{21}^{2}){]}^{2}}$$

One can see that $\frac{\partial {Z}_{eff}^{2}}{\partial {S}_{21}}$ approaches zero as |*S*_21_| → 0. Therefore, in our calculation we first determine *Z*_*eff*_ from *S*_11_ and a very small value of *S*_21_ (|*S*_21_| ≈ 0.001) using eq. (1). In addition, to find the correct sign of *Z*_*eff*_, we follow the method of Chen *et al*.^33^, which is based on a relation between *Z*_*eff*_ and *n*.

Once *Z*_*eff*_ is known, the exponential term ${e}^{in{k}_{0}d}$ can be calculated from the equation:

6 $${e}^{in{k}_{0}d}=\frac{{S}_{21}}{1-{S}_{11}\frac{{Z}_{eff}-1}{{Z}_{eff}+1}}$$

Substitution of eq. (6) in eq. (2) yields a unique value for the imaginary part *n*″ of the refractive index, but the real part *n*′ of the refractive index remains complicated due to the branches of the logarithm function. In order to precisely determine *n*′, one can use an iterative method based on a Taylor series in ref.^30^ or a method based on Kramers-Kronig relationship proposed by Szabó *et al*.^34^. Here, for simplicity, we calculated the absorption spectra for a variety of branches of *n*′ and found no significant differences. This indicates that the refractive index for small values of *S*_21_ has little influence on the resulting absorption spectra.

The resulting absorptivity is calculated through the total reflection (*R*_*tot*_) as *A* = 1 − *R*_*tot*_ while the total reflection is derived from the interference theory^35^:

7 $${R}_{tot}=\frac{{r}_{12}-{e}^{-2i\phi }}{1-{r}_{12}{e}^{-2i\phi }}\,$$

where *λ* is the wavelength of light in the free space. *φ* = (2*π*.*n*_*eff*_.*dcosθ*′)/*λ* are the maiden reflection from the equivalent substrate to the air and the propagation phase in the equivalent medium, respectively. If the incident light is normal to the air-effective medium interface (*θ* = 0), the angle of refraction is equal to zero (*θ*′ = 0) according to the Snell law.

**Figure 7:**
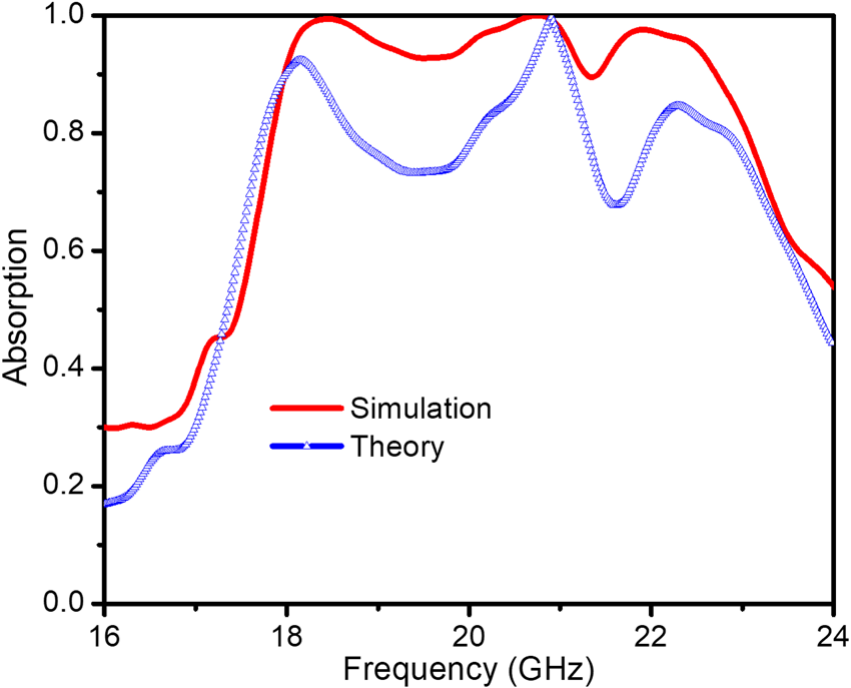
Comparison between numerical simulation (red line) and theoretical calculation (blue line) results.

The results of the theoretical calculation and the simulation with *θ* = 0 are shown in Fig. 7. The calculated result is in consensus with the simulated one and indicates clearly the broad absorption band from 18 to 23 GHz, with the absorptivity is over 80%. The equivalent medium interference theory is therefore adequate for explaining the absorption mechanism of our controlled-defect based metamaterial absorber structure. This result also confirms the validity of our method.

## Energy Loss in the Structure

**Figure 8:**
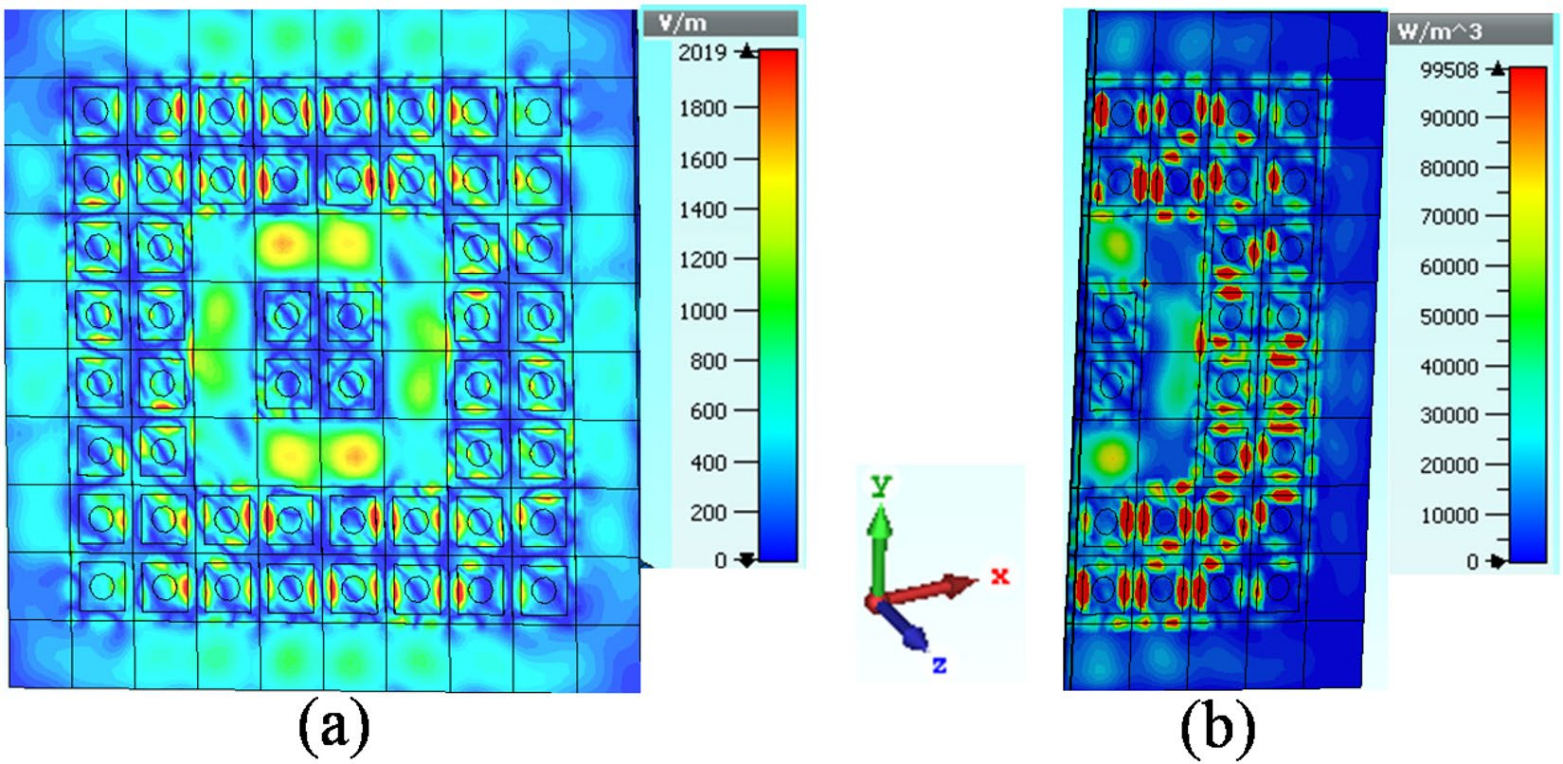
(**a**) Electric field distribution and (**b**) power loss density of the structure at frequency of 23 GHz.

To consider the underlying mechanism of energy distribution, we observe the electric field and power loss at 23 GHz (see Fig. 8), this is the fundamental absorption frequency in our study and it is chosen for studying the field in the range of absorption band. One can see that, the electromagnetic energy concentrated on the surface and at the defect region of the combined structure (Fig. 8a).

The distribution of the power loss density for the structure at 23 GHz are also observed (Fig. 8b). At the cross section, it shows that the loss energy concentrates mainly in the dielectric layer and at the defect region.

## Experiment Verification

**Figure 9:**
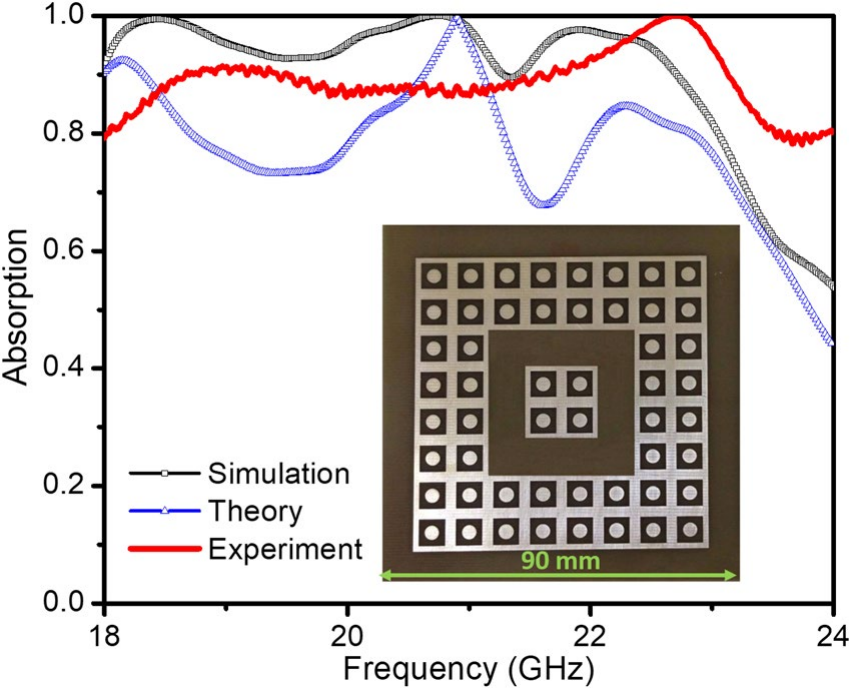
The experiment, calculation, and simulation results of absorption spectra of the combined structure. The inset shows fabricated prototype of the proposed combined metamaterial structure (in which the dimensions of one unit cell are *a* = 9 mm; *b* = 6.5 mm; *D* = 3.5 mm and *t* = 1.25 mm as shown in Fig. 1).

A prototype of optimal defect integrated full-sized structure (inset of Fig. 9) was fabricated by a standard photolithography method. The measurement is performed in an anechoic chamber using a vector network analyzer (Hewlett-Packard E8363B network analyzer). In detail, two linearly-polarized microwave standard-gain horn antennas were used for the illumination of the microwave beam on the sample and the reception of the reflected beam from the sample with an incident angle of 5 degrees (which can be regarded as at the normal incidence). In order to allow the EM waves to radiate sufficiently and to minimize the near-field effects, the distance between the midpoint of two horn antennas to the center of the sample is kept at approximately 100 times longer than the absorption wavelength, which is larger than the far-field of the antenna in the microwave anechoic chamber. For the same incident angle, the distance between the horn antennas and the sample does not influence the absorption spectrum if the far-field condition is fulfilled.

The absorption spectrum for the combined structure with 100 UCs after integrating with the optimal defect is presented in Fig. 9. The simulation, theory calculation, and measurement results are in a good agreement and indicate a fairly perfect absorption band from 18 to 23 GHz. However, the discrepancies in the frequency range and the absorption intensity are also observed. Frequency shifts and ripple levels in measurement results can be explained by the imperfections of our prototype, the installation of the horn antenna system, the inexact value of the sample substrate’s permittivity, and the possibility that the simulation may not have taken into account all parameters of the system.

## Polarization Insensitive Characteristic of the Absorber Structure

**Figure 10:**
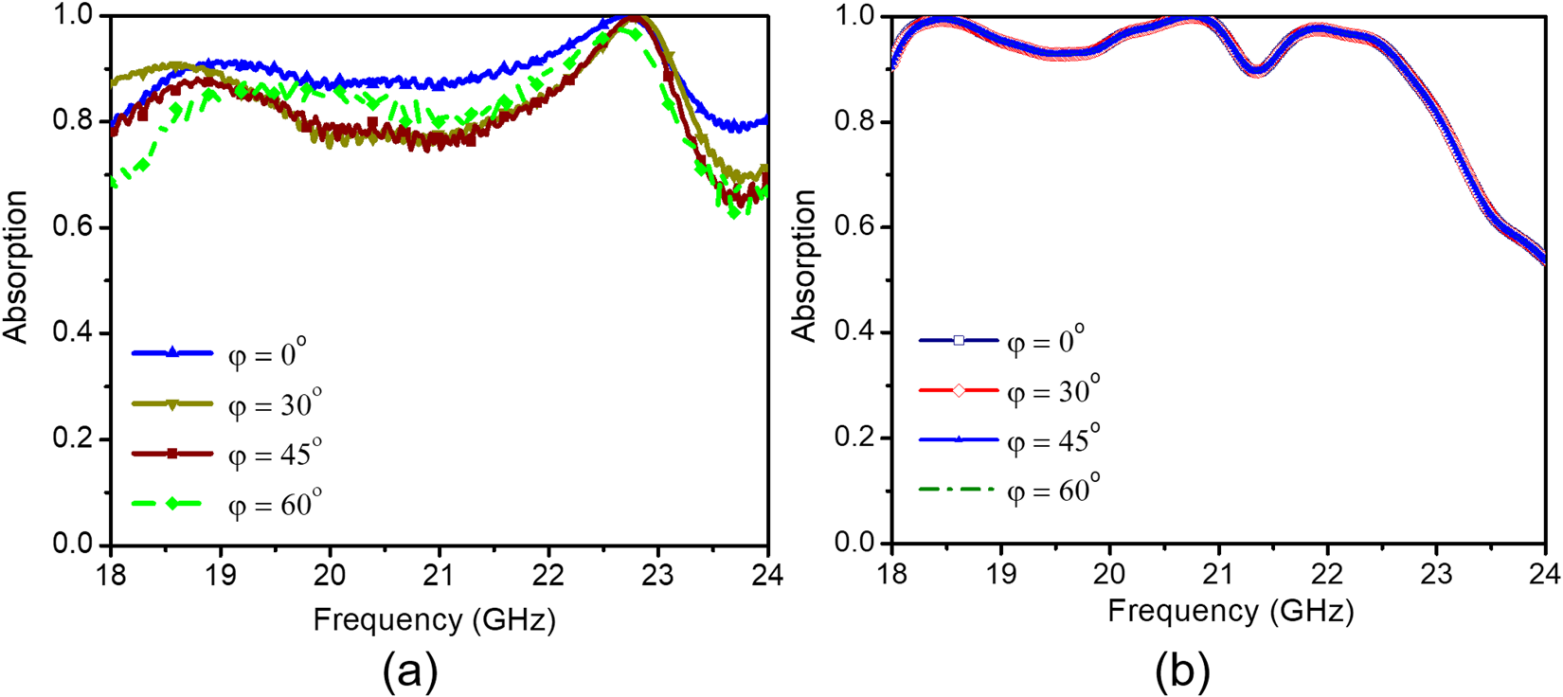
The (**a**) measurement and (**b**) simulation results of absorption spectra at different polarization angle.

The absorption spectra at different polarization angles (*φ*) of the incident wave for the optimal structure with 100 UCs are also presented in Fig. 10. The measurement results (Fig. 10a) and the simulation ones (Fig. 10b) are in a good agreement and indicate fairly the absorption band from 18 to 23 GHz. The measurement and simulation results also confirm that the absorption is independent of the polarization angle as expected.

## Structure in the THz Regime

**Figure 11:**
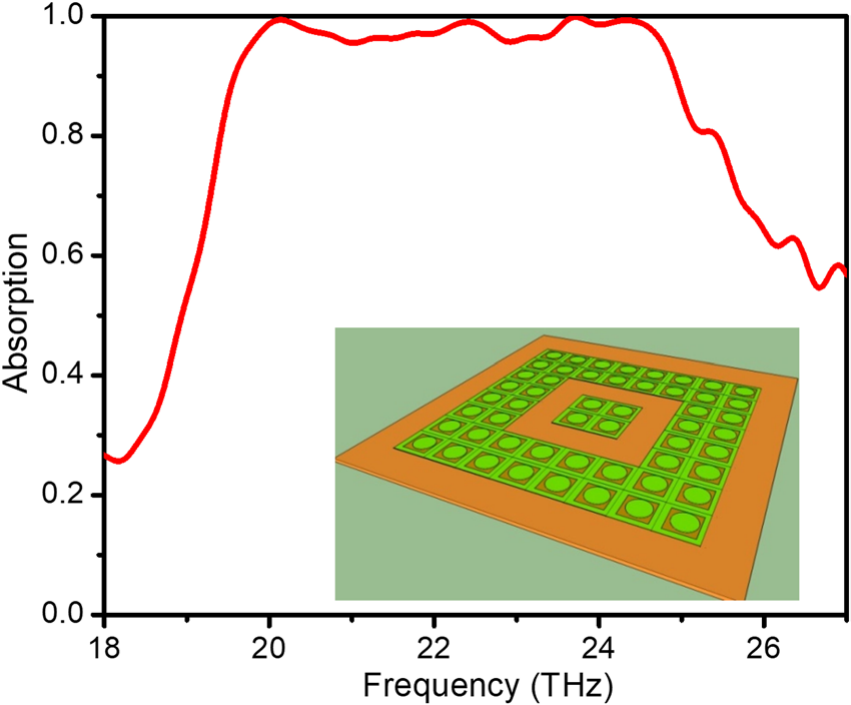
Absorption spectra of THz regime optimal structure. The inset shows the schematic image of the sample.

The THz technology possesses many momentous technological applications in imaging, sensing, recycling energy or in micro antenna communication, which all require perfect absorbers to efficiently collect wave energy. Therefore, metamaterial terahertz absorber especially the broadband MPA has various potential applications and has attracted the concern of many researchers^36–38^. The first MPA working at THz frequency was proposed by Tao *et al*. in 2008^36^. In this report, to enhance the applicability of the optimal defect structure to the THz frequency range, we extrapolate our study to have a broadband absorption at the THz scale. The structure dimension is shrunk down, the material of the two conductive layers is replaced by gold. The dielectric layer is polyimide with the dielectric constant is 3.4 + i0.025. For simplicity, the structural ratio of the unit cell is kept as in the GHz range case while the dimensions are all in micrometer scale. The area of the studied full structure is 90 × 90 µm^2^. The conditions of the excitation wave are kept unchanged. Simulation result shows that the wide absorption band appears in the range of 20–25 THz with a bandwidth of 5 THz (Fig. 11). The absorptivity reached over 95% in a wide frequency band. This structure can be readily manufactured using microfabrication technology. The results also show that the method of controlling the defects in metamaterial structure is practicable for the THz scale. This inaugurates a new way to study broadband terahertz metamaterial absorbers and their applications.

## Conclusions

The absorption characteristics of a new full-sized metamaterial absorber structure are numerically simulated, theoretically calculated and experimentally verified in GHz region then extrapolated in THz frequency. The results show that two ultra-broadbands, one of 5-GHz and one of 5-THz, with the absorption of over 95%, are obtained by optimizing and manipulating the defect walls. This has a great potential for microchips technology like MEMS (Microelectromechanical systems) or integrated micro-circuit, antenna systems, or sensors technology. Controlling defects is a simple way of tuning the electromagnetic responses of a particular absorber structure without the need of external interventions.

## Methods

### Simulation

Simulations are performed using the commercial software CST Studio Suite. To simulate the full-sized absorber structure, open boundary conditions are applied for the investigated configuration. A waveport is used for the excitation of the structure to obtain the S coefficients, the absorption then is deduced from the magnitude of S_11_ parameters.

### Theory calculation

By using a metal plane at the backplate, the final absorptivity is yielded through the total reflection (*R*_*tot*_) as *A* = 1 − *R*_*tot*_ while the total reflection is given from the real part and imaginary part of *S*_11_ *and S*_21_, which are extracted from the numerical simulation. Our Matlab® script was used for the data processing.

### Measurements

Measurements have been performed in an anechoic chamber using the Hewlett Packard E8363B 10 MHz to 40 GHz Series Vector Network Analyzer and a pair of standard horn antennas FR6517 (emission and reception), working in the range of 18–24 GHz. The reflection coefficient is normalized using a metal reflector at the backplate. The experimental results are plotted and compared with the simulation ones.
